# The overlap method is a safe and feasible for esophagojejunostomy after laparoscopic-assisted total gastrectomy

**DOI:** 10.1186/1477-7819-12-392

**Published:** 2014-12-20

**Authors:** Mamoru Morimoto, Hidehiko Kitagami, Tetsushi Hayakawa, Moritsugu Tanaka, Yoichi Matsuo, Hiromitsu Takeyama

**Affiliations:** Department of Surgery, Kariya Toyota General Hospital, Kariya, Japan; Department of Gastroenterological Surgery, Nagoya City University Graduate School of Medical Sciences, Kawasumi 1, Mizuho-cho, Mizuhoku, Nagoya, 467-8601 Japan

**Keywords:** Esophagojejunostomy, gastric cancer, laparoscopic surgery, total gastrectomy

## Abstract

**Background:**

Laparoscopic procedures are increasingly being applied to gastric cancer surgery, including total gastrectomy for tumors located in the upper gastric body. Even for expert surgeons, esophagojejunostomy after laparoscopy-assisted total gastrectomy (LATG) can be technically challenging. We perform the overlap method of esophagojejunostomy after LATG for gastric cancer. However, technical questions remain. Is the overlap method safer and more useful than other anastomosis techniques, such as methods using a circular stapler? In addition, while we perform this overlap reconstruction after LATG in a deep and narrow operative field, can the overlap method be performed safely regardless of body habitus? This study aimed to evaluate these issues retrospectively and to review the literature.

**Methods:**

From October 2005 to August 2013, we performed LATG with lymph-node dissection and Roux-en-Y reconstruction using the overlap method in 77 patients with gastric cancer. This study examined pre-, intra- and postoperative data.

**Results:**

Mean operation time, time to perform anastomosis, and estimated blood loss were 391.4 min, 36.3 min, and 146.9 ml, respectively. There were no deaths, and morbidity rate was 13%, including one patient (1%) who developed anastomotic stenosis. Mean postoperative hospitalization was 13.4 days. Surgical outcomes did not differ significantly by body mass index.

**Conclusions:**

First, the overlap method for esophagojejunostomy after LATG is safe and useful. Second, this method can be performed irrespective of the body type of the patient. In particular, in a deep and narrow operative field, the overlap method is more versatile than other anastomosis methods. We believe that the overlap method can become a standard reconstruction technique for esophagojejunostomy after LATG.

## Background

Laparoscopy-assisted distal gastrectomy for gastric cancer is a less invasive surgical procedure than open distal gastrectomy. Postoperative recovery is faster [1] and the procedure is more adaptable to patients with varying body habitus. In recent years, the use of laparoscopy-assisted distal gastrectomy has been gaining popularity in Japan and Korea [2, 3]. However, laparoscopy-assisted total gastrectomy (LATG) is not widely performed for gastric cancer. The reasons for this include the fact that laparoscopic lymph-node dissection is technically difficult, as is the creation of an esophagojejunostomy in a deep, narrow operative field. Anastomotic complications can be serious [4–9].

A standard method needs to be established for esophagojejunostomy to allow for safe performance irrespective of body habitus, in order to facilitate the adoption of LATG in the future. Various methods for esophagojejunostomy, including the purse-string sutured method with a hand-sewn technique or with other devices [10–21], OrVil™ (Covidien Japan, Tokyo, Japan) [22–33], functional end-to-end anastomosis (FEEA) [34–42] and the overlap method [43, 44], have been developed and techniques are surgeon-dependent. Having experience in several different methods, we believe that the overlap method represents the safest and most useful technique and could become the standard for esophagojejunostomy.

However, controversy remains regarding the safety and utility of the overlap method. Few reports have described the use of this method, and even fewer have made comparisons between this and other anastomotic techniques. The overlap method requires advanced suturing skill within the abdominal cavity, which is a deep and narrow operative field, and successful performance is dependent on the body type of the patient. This study provides the largest series of cases of anastomoses after LATG using the overlap method. The results of this retrospective study are discussed, with a literature review.

## Methods

### Patients

From October 2005 to August 2013, we performed LATG with lymph-node dissection according to Japanese Gastric Cancer Association guidelines [45] and Roux-en-Y reconstruction using the overlap method in 77 patients with gastric cancer at Kariya Toyota General Hospital. Informed consent was obtained from each patient prior to surgery. All operations were performed by two experienced laparoscopic surgeons (TH and HK) who have obtained endoscopic surgeon qualifications from Japan Society for Endoscopic Surgery, and have experience in over 100 laparoscopic-assisted gastrectomy cases. Neoadjuvant chemotherapy was not implemented in any of the cases.

Laparoscopy-assisted total gastrectomy for gastric cancer was indicated for preoperative stage T1 to 3, N0 to 1, M0 according to the Japanese Classification of Gastric Carcinoma (third English edition) [46].

We performed D1+ or D2 lymph-node dissection for all patients according to the Japanese Gastric Cancer Association guidelines [45]. D1+ lymph-node dissection is indicated for cT1N0 tumors other than those indication for D1 lymph-node dissection. D2 lymph-node dissection is indicated for potentially curable T2 or T3 tumors, as well as cT1N1 tumors. Furthermore, the indication for the overlap method is for tumors located at the cardia or within at least 2 to 3 cm below the esophagogastric junction, and not invading the esophagus. We could guarantee that the stapled line did not enclose the tumor in these cases.

During surgery, we do not place a jejunal feeding tube in the jejunum. The nasogastric tube was inserted preoperatively and removed on postoperative day (POD) 1, if there was no sign of bleeding from the staple line of the anastomosis. After the nasogastric tube was removed, the patients could drink clear fluids as desired. Routine swallow studies were performed in our hospital. Esophagojejunostomy was tested for patency or leakage via an upper gastrointestinal tract X-ray series with water-soluble contrast on POD 3. If there were no abnormal findings, the patients could begin oral intake of light rice gruel. Prophylactic intravenous antibiotics were continued until POD 3.

Clinical characteristics, such as intra- and postoperative data and pathological findings were retrospectively obtained from medical records. The ideal body mass index (BMI) for the Japanese is 22 kg/m^2^ [47], and incidentally the mean in our study was also 22 kg/m^2^. Because of this, to compare the impact of body type, the patients were divided into two groups; Group A had a BMI exceeding 22 kg/m^2^ and Group B had a BMI lower than 22 kg/m^2^. Surgical outcomes were compared between groups. Postoperative complications were classified according to the Clavien-Dindo classification of surgical complications [48].

### Surgical procedure

**Figure 1:**
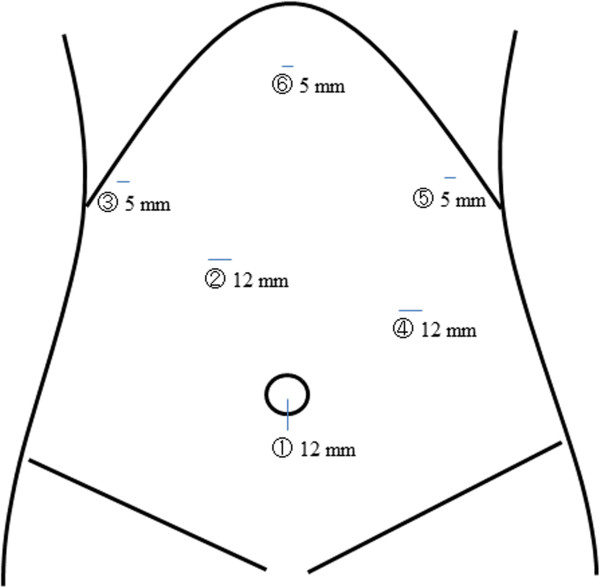
**Placement of trocars**. The first trocar is inserted at the umbilicus (1), and used in minilaparotomy. At (3) and (5), a 5-mm trocar is used. At (2) and (4), a 12-mm trocar is used. At (6), a liver retractor is used.

Under general anesthesia, patients were placed in the lithotomy position. The surgeon was positioned on the right side of the patient, the first assistant on the left side, and the laparoscopist between the abducted legs of the patient. A 12-mm camera port was inserted into a median umbilical incision. Pneumoperitoneum of 10 mmHg was induced, and four additional ports (two ports with a 12-mm diameter and two with a 5-mm diameter) were inserted under direct visualization in the upper abdomen (Figure 1). We exposed the abdominal esophagus and transected it at a line for which an adequate proximal margin could be obtained using a 60-mm endoscopic linear stapler. The resected stomach and surrounding fatty tissue, including retrieved lymph nodes, were placed in a plastic specimen bag. Before the reconstruction procedure, the specimen in the bag was retrieved through the extended umbilical port incision.

We perform the jejunojejunostomy (Y anastomosis) before the esophagojejunostomy. The jejunum was extracorporeally transected 20 cm distal to the ligament of Treitz using a 45-mm endoscopic linear stapler. The distal side of the jejunum (approximately 10 cm long) was sacrificed to avoid excessive tension at the site of anastomosis of the esophagojejunostomy. At the lumen 45 to 50 cm distal from the site for planned esophagojejunostomy, a side-to-side jejunojejunostomy was performed using a 45-mm endoscopic linear stapler. The entry hole was closed using an extracorporeal interrupted hand-sewn technique with absorbable monofilament sutures. After suturing the umbilical incision to the size of the trocar, pneumoperitoneum was re-established.

**Figure 2:**
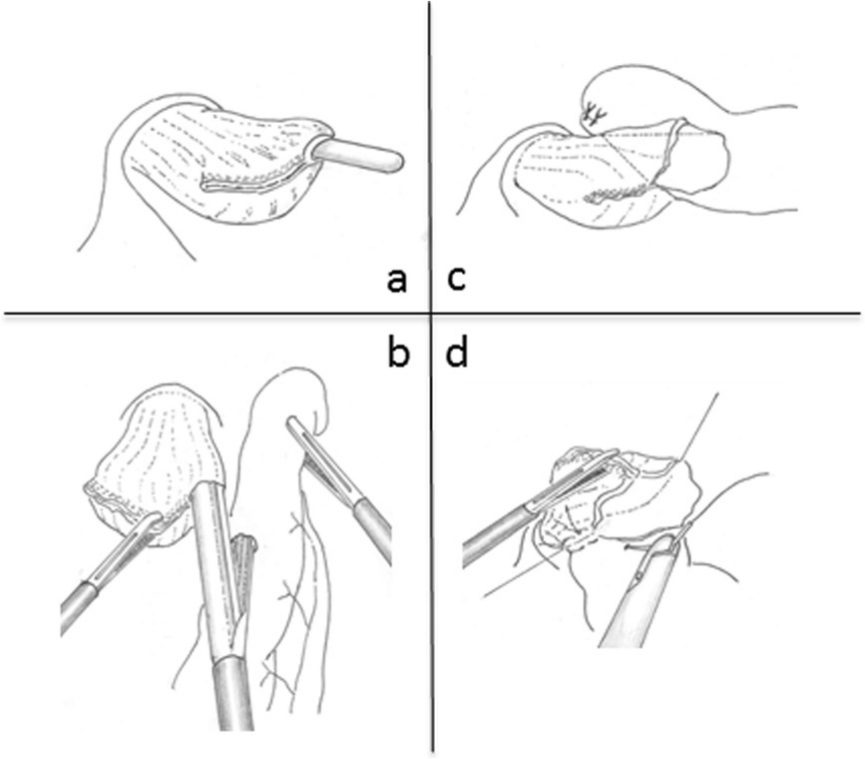
**Schema of the overlap method. (a)** A small opening is made on the left wall of the esophageal stump. **(b)** An endoscopic linear stapler is applied between the esophageal stump and the jejunal limb. **(c)** An anastomotic staple line is created between the esophagus and jejunum. **(d)** The entry hole is closed using an intracorporeal interrupted hand-sewn technique.

A small enterotomy was made 5 cm distal to the stapler line on the antimesenteric side of the jejunal limb, while another small enterotomy was made on the left wall of the esophageal stump. We inserted a nasogastric tube into the abdominal cavity via the small enterotomy of the esophageal stump (Figure 2a). After the anvil fork of the 45-mm endoscopic linear stapler was inserted into the opening made in the jejunal limb toward the oral side of the lumen, the jejunal limb was drawn up and positioned at the left side of the abdominal esophagus to create an esophagojejunostomy in an antecolic fashion. The cartridge fork of the linear stapler was inserted into the opening made in the esophageal stump (Figure 2b). After each fork was completely inserted into each lumen, the two limbs were joined together to fashion a side-to-side esophagojejunal anastomosis. The firing of the stapler converted the two openings into a single entry hole to create an esophagojejunostomy; intraluminal hemostasis was then confirmed (Figure 2c).

The entry hole of the stapler was closed using an intracorporeal interrupted hand-sewn technique combined with Roeder’s knots, an extracorporeal ligature technique. Absorbable monofilament suture was used for suturing. At first, we sutured at both ends of the hole, and pulled the thread to the opposite side as an anchor suture. The entry hole was rotated 90°, so that it becomes easier to securely close the entry hole by hand-sewn suturing in the narrow and deep field under laparoscopy (Figure 2d). Interrupted sutures through all layers were usually placed 10 to 12 times, and as a result the intracorporeal esophagojejunostomy was completely established. After completing the intracorporeal Roux-en-Y reconstruction, three stitches were placed in the duodenal stump and the antimesenteric side of the jejunal limb to prevent the limb from falling into the left dorsal subphrenic space and kinking of the esophagojejunal anastomosis.

### Statistical analysis

All values are presented as mean ± standard deviation. Statistical analysis was performed using the Mann–Whitney *U* test, chi-square test, or unpaired Student’s *t* test. All *P* values were two-sided and those of 0.05 or less were considered statistically significant. All statistical analyses were performed using EZR [49], a graphical user interface for R (The R Foundation for Statistical Computing, Vienna, Austria, version 2.13.0). More precisely, EZR is a modified version of R Commander (version 1.6-3) that was designed to add statistical functions frequently used in biostatistics.

## Results

### Clinical characteristics

The clinical characteristics of participants in this study are listed in Table 1. The mean age of patients was 66.2 years (range, 34 to 89 years), and 61 of the 77 patients were male. Thirty-seven patients (48%) had at least one comorbidity. The mean BMI was 22.4 kg/m^2^ (range, 16.5 to 29.3 kg/m^2^). We performed D2 dissection for 28 patients (36%) and D1+ dissection for 49 patients (64%).

**Table 1:** **Clinical characteristics**

Age (years)		66.2 ± 12.2 (34 to 89)
Sex (male: female)		61:16
Body mass index (kg/m^2^)		22.4 ± 2.9 (16.5 to 29.3)
American Society of Anesthesiology	1	17 (22%)
	2	60 (78%)
Concurrent illness*	No	40 (52%)
	Yes	37 (48%)
Cardiovascular disease		23
Diabetes mellitus		4
Respiratory disease		2
Liver disease		1
Other operation		13
Brain disorder		3
Extent of lymph-node dissection		
D1+		49 (64%)
D2		28 (36%)

*Some patients had more than one comorbidity.

### Operative and postoperative data

Operative and postoperative data from this study are listed in Table 2. Mean operation time, time to perform anastomosis, and estimated blood loss were 391.4 min (range, 280 to 495 min), 36.3 min (range, 24 to 52 min), and 146.9 ml (range, 3 to 510 min), respectively. No cases required conversion to open surgery. The mean number of harvested lymph nodes in all patients, D1+ and D2 lymph-node dissection were 40.5 (range, 16 to 83), 38 (range, 16 to 65) and 42 (range, 17 to 83), respectively. Mean postoperative hospitalization was 13.4 days (range, 7 to 96 days). The mortality rate was 0%, while postoperative complications were observed in 10 patients (13%). Anastomotic stenosis was observed in 1 patient (1%), which improved with conservative treatment without endoscopic balloon dilatation. We deemed this patient as grade I according to the Clavien-Dindo classification [47], and he was discharged from hospital on POD 12. Pancreatitis occurred in four patients (5%), all of whom recovered with conservative therapy without any intervention. These four patients were discharged on PODs 12, 32, 16 and 19. Intra-abdominal bleeding was observed in two patients (3%). Both patients required reoperation, and surgical complication was deemed as grade IIIb according to the Clavien-Dindo classification. They were discharged on PODs 10 and 96. Duodenal stump leakage occurred in two patients (3%), for whom surgical complication was deemed as grade IIIa according to the Clavien-Dindo classification. Discharge from hospital was on PODs 27 and 38, respectively. An intra-abdominal abscess was observed in one patient (1%), whose surgical complication was grade IIIa according to the Clavien-Dindo classification, and who was discharged on POD 23.

**Table 2:** **Operative and postoperative data**

Operation time (min)		391.4 ± 51.3 (280 to 495)	
Time to perform anastomosis (min)		36.3 ± 6.8 (24 to 52)	
Estimated blood loss (ml)		146.9 ± 129.5 (3 to 510)	
Transfusion		1 (1%)	
Conversion to open surgery		0	
Number of harvested lymph nodes		40.5 ± 13.7 (16 to 83)	
	D1+	38 ± 12.7 (16 to 65)	
	D2	42 ± 14.1 (17 to 83)	
Combined resection	Spleen	23 (30%)	
	Gall bladder	4 (5%)	
Time until start of oral intake (days)		3.9 ± 2.1 (2 to 17)	
Time to first flatus (days)		2.3 ± 0.9 (1 to 4)	
Postoperative hospital stay (days)		13.4 ± 5.8 (7 to 96)	
Complications	No	68 (88%)	
	Yes	10 (13%)	
Complications related to anastomosis		1 (1%)	
	Leakage	0	
	Bleeding	0	
	Stenosis	1 (1%)	Grade I*
Pancreatitis		4 (5%)	
Intra-abdominal bleeding		2 (3%)	Grade IIIb*
Duodenal stump leakage		2 (3%)	Grade IIIa*
Intra-abdominal abscess		1 (1%)	Grade IIIa*

*According to the Clavien-Dindo classification of surgical complications.

### Pathological findings

Pathological data according to the Japanese Classification of Gastric Carcinoma (third English edition) [46] are listed in Table 3. The mean proximal resected margin was 21.1 mm (range, 10 to 35 mm). No residual cancer cells were present at the cut edges of the esophagus. Pathological stages IA, IB, IIA, IIB, IIIA, and IIIB made up 39%, 14%, 16%, 8%, 12%, and 12%, respectively, of the total number of carcinomas. All patients had R0 operations.

**Table 3:** **Pathological findings**

Histological type	
Well differentiated	12 (16%)
Moderately differentiated	27 (35%)
Poorly differentiated	31 (40%)
Signet ring cell	4 (5%)
Other (med, pap)	3 (4%)
Proximal resected margin (mm)	21.1 ± 9.7 (10 to 35)
Stage*	
IA	30 (39%)
IB	11 (14%)
IIA	12 (16%)
IIB	6 (8%)
IIIA	9 (12%)
IIIB	9 (12%)

*According to the Japanese Classification of Gastric Carcinoma: 3rd English Edition.

### Surgical outcomes according to body mass index

We applied BMI 22 kg/m^2^ as a cut-off to classify cases (Group A, BMI > 22 kg/m^2^; Group B, BMI < 22 kg/m^2^. Surgical outcomes for both groups are listed in Table 4. In Group A, mean operation time, time to perform anastomosis, and estimated blood loss were 392.8 min (range, 280 to 495 min), 37.0 min (range, 24 to 52 min), and 156.0 ml (range, 5 to 510 ml), respectively. Postoperative complications were observed in six patients (13%). Complications related to anastomosis were not encountered. In Group B, mean operation time, time to perform anastomosis, and estimated blood loss were 389.2 min (range, 285 to 464 min), 35.4 min (range, 28 to 44 min), and 133.2 ml (range, 10 to 360 min), respectively. Postoperative complications were observed in four patients (13%). Anastomotic stenosis was observed in one patient (1%). No significant differences were identified between the two groups.

**Table 4:** **Surgical outcomes (BMI: 22 kg/m** ^**2**^ **)**

		Group A (***n*** = 47)	Group B (***n*** = 30)	***P***
Operation time (min)		392.8 ± 55.1 (280 to 495)	389.2 ± 45.7 (285 to 464)	—
Time to perform anastomosis (min)		37.0 ± 7.7 (24 to 52)	35.4 ± 5.6 (28 to 44)	—
Estimated blood loss (ml)		156.0 ± 138.8 (5 to 510)	133.2 ± 115.1 (10 to 360)	—
Complications	No	41 (87%)	26 (87%)	—
	Yes	6 (13%)	4 (13%)	—
Complications related to ansastomosis		0	1 (3%)	—
	Leakage	0	0	—
	Bleeding	0	0	—
	Stenosis	0	1 (3%) Grade I*	—
Pancreatitis		2 (4%)	2 (7%)	—
Intra-abdominal bleeding		2 (4%) Grade IIIb*	0	—
Duodenal stump leakage		2 (4%) Grade IIIa*	0	—
Intra-abdominal abscess		0	1 (3%) Grade IIIa*	—

*According to the Clavien-Dindo classification of surgical complications. —, not significant.

## Discussion

This study made two important clinical observations. First, the overlap method for esophagojejunostomy after LATG is safe and useful. Second, this method can be performed irrespective of the body type of the patient. We can perform gastrojejunostomy and gastroduodenostomy during laparoscopy-assisted distal gastrectomy in a wide visual field. However, esophagojejunostomy after LATG is performed in a deep and narrow field between the crura of the diaphragm. Reconstruction after LATG is a complicated procedure and the visual field is particularly narrow in obese patients. This probably accounts for the higher rate of anastomotic complications after LATG.

Complications related to anastomoses are potentially very serious [4–9]. This issue may therefore interfere with the wider adoption of LATG. To help the spread of LATG, a standard esophagojejunostomy method that is not difficult, technically complicated, or influenced by the body type of the patient and has few complications related to anastomosis should be established. Various reports have described esophagojejunostomy using circular-stapled anastomosis and linear-stapled anastomosis. Circular-stapled anastomosis includes purse-string sutured methods with a hand-sewn technique or with other devices [10–21] and esophagojejunostomy using OrVil™ (Covidien Japan, Tokyo, Japan) [22–33], while FEEA [34–42] and the overlap method [43, 44] are both categorized as linear-stapled anastomosis. The results of this study are comparable to those of previous studies in terms of surgical outcomes, including time to perform anastomosis, blood loss, duration of postoperative hospitalization, and frequencies of leakage, stenosis, and mortality (Tables 5 and 6).

**Table 5:** **Previous reports of intracorporeal anastomosis using circular stapler in LATG**

Reference	Year	Number of patients	Body mass index (kg/m ^2^)	Mortality	Operation time (min)	Time to perform anastomosis (min)	Blood loss (ml)	Hospitalization (days)	Complications	Anastomotic stenosis	Anastomotic leakage
**Purse-string sutured by hand or with other devices**											
[10]	2005	8	—	0%	—	—	—	—	—	—	12.5%
[11]	2005	8	—	0%	183	—	81	16.9	13%	0%	0%
[12]	2005	10	—	0%	—	—	—	—	10%	0%	10%
[13]	2006	63	—	—	—	—	—	—	—	0%	4.8%
[14]	2008	27	22.6	0%	527.5	—	—	16.2	7%	—	0%
[15]	2008	20	—	0%	254	—	299	19	25%	5%	10%
[16]	2008	38	24.0	2.6%	187	—	10	—	39%	—	5.3%
[17]	2008	23	23.4	0%	305.9	—	77.5	11.2	4%	0%	0%
[18]	2009	16	—	6.3%	225	—	—	16	—	0%	6.3%
[19]	2009	67	22.9	0%	305.4	—	190.7	13.6	27%	9%	1.5%
[20]	2010	10	22.4	0%	257	—	69	13	10%	0%	0%
[21]	2013	100	—	0%	249	—	182	—	18%	—	6%
**Orvil™**											
[22]	2009	16	23.0	0%	194	—	272	11	6%	0%	0%
[23]	2010	27	24.0	0%	—	—	—	—	4%	3.8%	0%
[24]	2011	30	23.0	3.3%	209.8	64.5	111	21.9	7%	—	3.3%
[25]	2011	16	24.9	0%	—	—	—	—	44%	18.8%	0%
[26]	2012	13	—	8.6%	—	—	—	—	15%	7.5%	0%
[27]	2013	12	24.3	0%	226.5	42.8	—	8.4	—	33.3%	16.7%
[28]	2013	16	—	0%	—	—	—	—	25%	—	0%
[29]	2013	40	24.0	0%	220.2	18.6	—	11.6	—	3%	5%
[30]	2013	21	21.2	0%	198	—	130	12.5	—	5%	5%
[31]	2013	28	—	0%	143	—	70	9.6	7%	0%	—
[32]	2013	17	27.1	2%	—	—	—	—	31%	5.9%	5.9%
[33]	2013	52	22.8	0%	—	—	—	—	—	21%	1.9%

—,not recorded.

**Table 6:** **Previous reports of intracorporeal anastomosis using linear stapler in LATG**

Authors	Year	Number of patients	Body mass index (kg/m ^2^)	Mortality	Operation time (min)	Time to perform anastomosis (min)	Blood loss (ml)	Hospitalization (days)	Complications	Anastomotic stenosis	Anastomotic leakage
**Functional end-to-end anastomosis**											
[34]	1999	2	—	0%	595	—	367.5	29.5	—	0%	0%
[35]	2002	3	—	33%	—	—	—	—	33%	0%	0%
[36]	2008	4	—	0%	381	86	313	11	0%	0%	0%
[37]	2008	14	—	0%	255.1	42.5	107.5	—	—	0%	0%
[38]	2009	15	20.8	0%	325	—	195	11	13%	0%	0%
[39]	2009	55	—	0%	406	—	102	17	33	—	3.6%
[40]	2010	56	—	1.5%	249	44	—	12.4	29%	3%	6%
[41]	2012	27	24.6	0%	126	—	—	8.1	11%	0%	0%
[42]	2013	65	23.5	1.5%	271.5	—	85.2	21.4	15.4%	4.6%	0%
**Overlap method**											
[43]	2010	53	22.0	0%	373.4	—	146.5	14.4	24.5%	0%	3.8%
[44]	2012	15	21.7	0%	236.4	—	51.2	13.5	16%	0%	0%
**This study**	*2013*	*77*	*22.4*	*0%*	*391.4*	*36.3*	*146.9*	*13.4*	*13%*	*1%*	*0%*

—, not recorded.

We believe that the overlap method can be performed more safely than other methods of anastomosis, for many reasons. One report notes that the blood supply to the staple line after linear-stapled anastomosis does not fall to critical levels [50], leading to a lower risk of anastomotic leakage. As a linear stapler is thinner and has better mobility in the tip than a circular stapler, handling of the linear stapler is easier than that of a circular stapler in the deep and narrow field of LATG. Moreover, compared with circular-stapled anastomosis, the use of linear-stapled anastomosis allows the surgeon to avoid torsion of the jejunal limb and involution of other organs that might lead to complications related to the anastomosis. In addition, linear-stapled anastomosis can be performed regardless of esophageal caliber and results in a larger anastomotic caliber than in circular-stapled anastomosis. When the esophageal caliber is small, it is necessary to use a smaller-caliber circular stapler in circular-stapled anastomosis. The use of a 21-mm circular stapler and the double-stapling technique is reportedly a risk factor for anastomotic stenosis [33]. Furthermore, we think that the overlap method is more useful than FEEA. The reasons are as follows. While FEEA is a simple, easy anastomosis method that can be performed in a relatively short time, there are two main differences between FEEA and the overlap method. The first is the peristaltic direction of the esophagojejunostomy. Because FEEA is performed in an anti-peristalsis direction in esophagojejunostomy, there is a need to lift the jejunal limb further up than for the overlap method. When the patient has a large amount of fat in the abdominal cavity, mobilization of the jejunum is needed to avoid tension on the jejunal limb that might result in anastomotic leakage. The second is that all anastomotic procedures are performed using linear staplers in FEEA, which needs a larger working space than the overlap method to close the entry hole of the anastomosis using linear staplers. To secure this large working space, a large incision of the crura of the diaphragm must be made in FEEA, and this may sometimes lead to a diaphragmatic hernia. For these reasons, we have adopted the overlap method as the first choice for reconstruction after LATG. This study compared the overlap method and other reported methods of anastomosis in terms of mean time to perform anastomosis and complications related to anastomosis. The mean time to perform anastomosis with purse-string sutured methods using a hand-sewn technique or with other devices was not mentioned. Times to anastomosis with OrVil™ and FEEA were 18.6 to 64.5 min and 44 to 86 min, respectively. Rates of anastomotic stenosis in the purse-string sutured methods, OrVil™ and FEEA were 0 to 9%, 0 to 33.3%, and 0 to 4.6%, respectively. Anastomotic leakage was observed in 0 to 12.5% of reports using purse-string sutured methods, 0 to 16.7% of OrVil™, and 0 to 6% of FEEA, respectively. The mean time to perform anastomosis was 36.3 min for the overlap method that we performed, a relatively short time compared with other reports. With the overlap method used in this study, anastomotic stenosis and leakage occurred in only 1% and 0% of cases, respectively, representing satisfactory outcomes.

The overlap method requires relatively advanced suturing skills in the abdominal cavity and is generally thought to prolong the time to perform anastomosis and increase stress levels for surgeons. Nevertheless, the time to perform anastomosis in this study was rather short compared with other methods. We thought this might be because we used Roeder’s knot, the extracorporeal ligature technique. There are two ligature techniques, intra- and extracorporeal. Because it is difficult to use needle and thread in a narrow and deep field around the esophagojejunostomy, we reasoned that the extracorporeal ligature technique could be performed more easily than the intracorporeal ligature technique.

## Conclusions

Although, there is a need to practice suturing skills in the abdominal cavity, perform preoperative simulation, and arrange for cooperation between operating room staff, the method does lead to a low incidence of complications. Furthermore, comparison of outcomes based on BMI revealed no significant difference between groups. We conclude that the overlap method is as safe and useful as other methods of anastomosis. We believe that the overlap method can become a standard technique for esophagojejunostomy after LATG.

Currently, the overlap method is a specific technique after LATG in our hospital. In future, we will perform the overlap method after open total gastrectomy. We plan to compare the overlap method after open total gastrectomy with other anastomotic methods. In addition, we think that a prospective, randomized, controlled trial is essential to obtain definitive evidence with regard to the standard procedure for esophagojejunostomy after LATG.

## Abbreviations

BMI: body mass index
FEEA: functional end-to-end anastomosis
LATG: laparoscopy-assisted total gastrectomy
POD: postoperative day.
